# Salivary Aldosterone as a Biomarker for Obstructive Sleep Apnea: Diagnostic Performance and the Moderating Role of Mean Arterial Pressure—A Cross-Sectional Study

**DOI:** 10.3390/clockssleep8030047

**Published:** 2026-08-17

**Authors:** Ziyuan Chen, May Nak Lau, Tengku Nurfarhana Nadirah Tengku Hamzah, Aida Nur Ashikin Abd Rahman, Liang Chye Goh, Mang Chek Wey

**Affiliations:** 1 Department of Orthodontics, Paediatric and Special Care Dentistry, Faculty of Dentistry, Universiti Malaya, Kuala Lumpur 50603, Malaysia; s2176337@siswa.um.edu.my (Z.C.); minalau@um.edu.my (M.N.L.); tengkunurfarhana@um.edu.my (T.N.N.T.H.); 2 Centre for Paediatric Dentistry & Orthodontic Studies, Faculty of Dentistry, Universiti Teknologi MARA, Jalan Hospital, Sungai Buloh 47000, Malaysia; aida_nurashikin@uitm.edu.my; 3 Department of Otorhinolaryngology, Faculty of Medicine, Universiti Malaya, Kuala Lumpur 50603, Malaysia

**Keywords:** sleep apnea syndromes, aldosterone, saliva, biomarkers, diagnostic accuracy, mean arterial pressure

## Abstract

Obstructive sleep apnea (OSA) is associated with renin–angiotensin–aldosterone system (RAAS) dysregulation. This study examined the relationship between salivary aldosterone and OSA severity, identified predictors of salivary aldosterone, evaluated the moderating role of mean arterial pressure (MAP), and compared the diagnostic performance of salivary aldosterone with the STOP-BANG questionnaire. This cross-sectional study included 67 adults grouped as healthy or mild OSA (apnea–hypopnea index [AHI] < 15; *n* = 34) versus moderate-to-severe OSA (AHI ≥ 15; *n* = 33). Morning salivary aldosterone was measured using enzyme-linked immunosorbent assay. Generalized linear models assessed independent predictors, MAP moderation was tested using PROCESS Model 1, and diagnostic performance was evaluated using receiver operating characteristic analysis. Salivary aldosterone did not differ between OSA severity groups and showed poor diagnostic performance. Age was negatively associated with aldosterone levels, while MAP moderated the relationship between AHI and salivary aldosterone. In contrast, the STOP-BANG questionnaire demonstrated acceptable discriminative ability and significantly higher specificity. Salivary aldosterone is not a reliable standalone OSA screening biomarker. However, MAP moderated the OSA–aldosterone relationship, suggesting potential utility in OSA patients with elevated MAP. Age independently predicted lower aldosterone, likely reflecting age-related RAAS decline. These findings support selective utility of salivary aldosterone in OSA patients with elevated blood pressure.

## 1. Introduction

Obstructive sleep apnea (OSA) is a common sleep-related breathing disorder characterized by recurrent upper airway obstruction during sleep, resulting in intermittent hypoxemia, sleep fragmentation, sympathetic activation, and intrathoracic pressure swing [1,2]. These pathophysiological changes contribute to substantial cardiovascular and metabolic morbidity, as well as accident risk [3,4,5]. Prevalence in adults ranges from 9% to 38% [6,7,8], yet the majority of affected individuals remain undiagnosed. The clinical consequences of untreated OSA—including hypertension, cardiovascular disease, stroke, and metabolic dysfunction—underscore the urgency of early and accurate diagnosis [9].

Polysomnography (PSG) remains the gold standard for OSA diagnosis but is costly, labor-intensive, and inaccessible in resource-limited settings. Validated clinical tools such as the STOP-BANG questionnaire offer practical screening alternatives, though their reliance on subjective self-report limits diagnostic precision. Consequently, there is growing interest in identifying objective, non-invasive biomarkers to facilitate scalable OSA screening [10]. Several salivary biomarkers have been evaluated for OSA [11], and systemic inflammatory markers including Tumor Necrosis Factor-alpha, C-reactive Protein, and interleukins have been explored in relation to OSA severity [12]. However, the diagnostic utility of these biomarkers remains limited owing to inconsistent findings and lack of specificity [13].

Aldosterone, a key effector hormone of the renin–angiotensin–aldosterone system (RAAS), regulates blood pressure, fluid balance, and sympathetic tone [14]. RAAS dysregulation has been implicated in OSA, as chronic intermittent hypoxia and heightened sympathetic activation may stimulate aldosterone secretion, contributing to the hypertension frequently observed in OSA patients [14,15,16,17]. Studies have reported elevated plasma aldosterone in OSA [18,19], though findings have been inconsistent, particularly in normotensive populations. Salivary aldosterone, which correlates closely with plasma free aldosterone [20,21,22], offers a non-invasive means of measurement and has been proposed as a candidate OSA biomarker. However, its diagnostic accuracy in OSA remains to be established.

Beyond diagnostic performance, the interplay between OSA, blood pressure, and RAAS activity warrants investigation. Mean arterial pressure (MAP) is a physiologically relevant integrator of blood pressure load and may modulate RAAS responsiveness in OSA. To date, no study has formally examined MAP as a moderator of the OSA–aldosterone relationship using salivary sampling.

This study aimed to: (1) examine whether salivary aldosterone concentrations differ across OSA severity groups; (2) identify independent clinical predictors of salivary aldosterone; (3) determine whether MAP moderates the relationship between OSA severity and salivary aldosterone; and (4) evaluate and compare the diagnostic accuracy of salivary aldosterone and the STOP-BANG questionnaire for identifying moderate-to-severe OSA.

## 2. Results

### 2.1. Participant Flow and Baseline Characteristics

**Figure 1:**
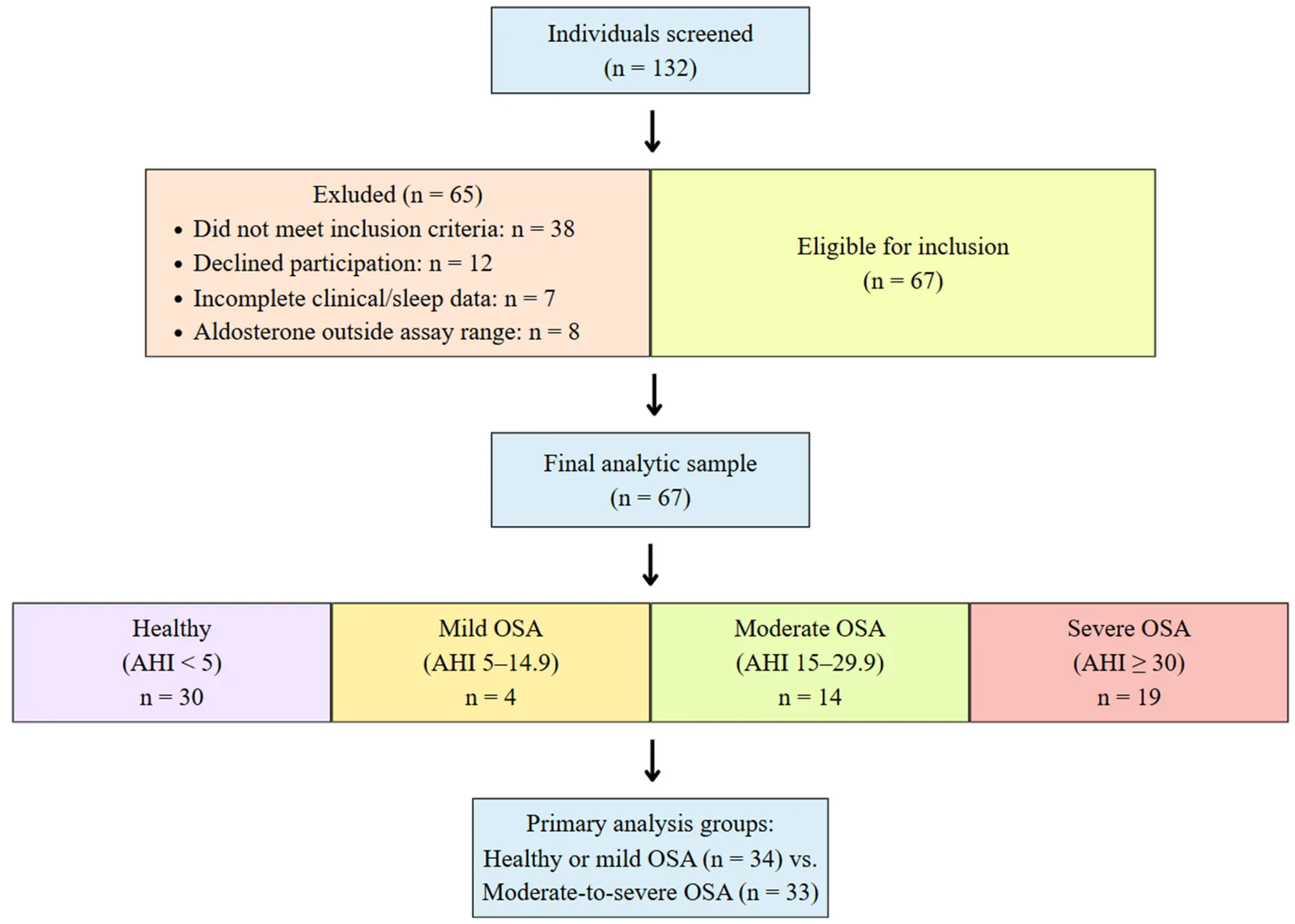
Flow diagram illustrating participant screening, eligibility assessment, and inclusion in the final analysis. AHI, apnea–hypopnea index; OSA, obstructive sleep apnea.

A total of 132 individuals were screened; 65 were excluded (did not meet inclusion criteria: *n* = 38 declined participations; *n* = 12 had incomplete data; *n* = 7 had aldosterone values outside assay range: *n* = 8), yielding a final sample of 67 participants (Figure 1). 

The study population comprised 30 healthy controls (44.8%), 4 individuals with mild OSA (6.0%), 14 with moderate OSA (20.9%), and 19 with severe OSA (28.4%). Among participants with OSA, the mean apnea–hypopnea index (AHI) values were 2.2 ± 1.5 events/h for mild OSA, 13.0 ± 2.0 events/h for moderate OSA, and 55.3 ± 19.4 events/h for severe OSA. Sociodemographic and clinical characteristics are summarized in Table 1.

Compared with the healthy or mild OSA group, participants with moderate-to-severe OSA were significantly older (*p* < 0.001) and exhibited higher BMI (*p* < 0.001), SBP (*p* < 0.001), DBP (*p* = 0.018), MAP (*p* = 0.002), and NC (*p* = 0.003). The ODI was also significantly higher in the moderate-to-severe OSA group compared with the healthy or mild OSA group (*p* < 0.001), whereas the lowest SpO_2_ was significantly lower in the moderate-to-severe OSA group (*p* < 0.001). No significant difference was observed in sex distribution between the two groups (*p* = 0.901). Compared with the healthy or mild OSA group, participants with moderate-to-severe OSA had a higher prevalence of hypertension (*p* < 0.001) and antihypertensive medication use (*p* = 0.001). No significant between-group differences were observed in dyslipidemia (*p* = 0.114), cardiovascular disease (*p* = 0.493), cerebrovascular disease (*p* = 0.493), ARB use (*p* = 0.239), or statin use (*p* = 0.114).

### 2.2. Salivary Aldosterone Concentrations Across Groups

Morning salivary aldosterone concentrations did not differ significantly between the moderate-to-severe OSA group and the healthy or mild OSA group (68.54 [34.98–293.30] vs. 68.35 [41.15–510.50], *p* = 0.821; Table 2).

In the generalized linear model (GLM) adjusting for age, BMI, NC, and MAP, OSA severity (moderate-to-severe vs. healthy or mild) remained a non-significant predictor of salivary aldosterone (B = 0.02; 95% CI −0.935 to 0.975; *p* = 0.968). Of all covariates examined, only age emerged as a significant independent predictor (B = −0.035; 95% CI −0.062 to −0.009; *p* = 0.010), with higher age inversely associated with salivary aldosterone levels (Table 3). BMI, NC, and MAP were not independently significant in the adjusted model (all *p* > 0.05).

### 2.3. Supplementary Analysis Using AHI as a Continuous Variable

In a supplementary multiple linear regression analysis, AHI was modelled as a continuous variable to further assess the association between OSA severity and salivary aldosterone levels. After adjustment for age, BMI, NC, and MAP, AHI was not significantly associated with log-transformed salivary aldosterone levels (B = −0.004, 95% CI −0.012 to 0.003, *p* = 0.252) (Supplementary Table S1).

### 2.4. Moderating Role of MAP in the OSA–Aldosterone Relationship

Moderation analysis using PROCESS macro-Model 1, adjusted for age, BMI, and NC (*n* = 67), revealed that MAP significantly moderated the relationship between OSA severity and log-transformed salivary aldosterone (interaction term b = 0.0203; *p* = 0.044).

At the mean level of MAP, the main effect of OSA severity on aldosterone was not significant (b = −0.011; *p* = 0.957). However, within the moderate-to-severe OSA group, MAP was positively and significantly associated with log-aldosterone (b = 0.0167; *p* = 0.013), corresponding to an approximately 18% increase in salivary aldosterone per 10 mmHg increment in MAP. In contrast, this relationship was absent in the healthy or mild OSA group (b = 0.006; *p* = 0.206).

### 2.5. Diagnostic Accuracy and Comparison with STOP-BANG

**Figure 2:**
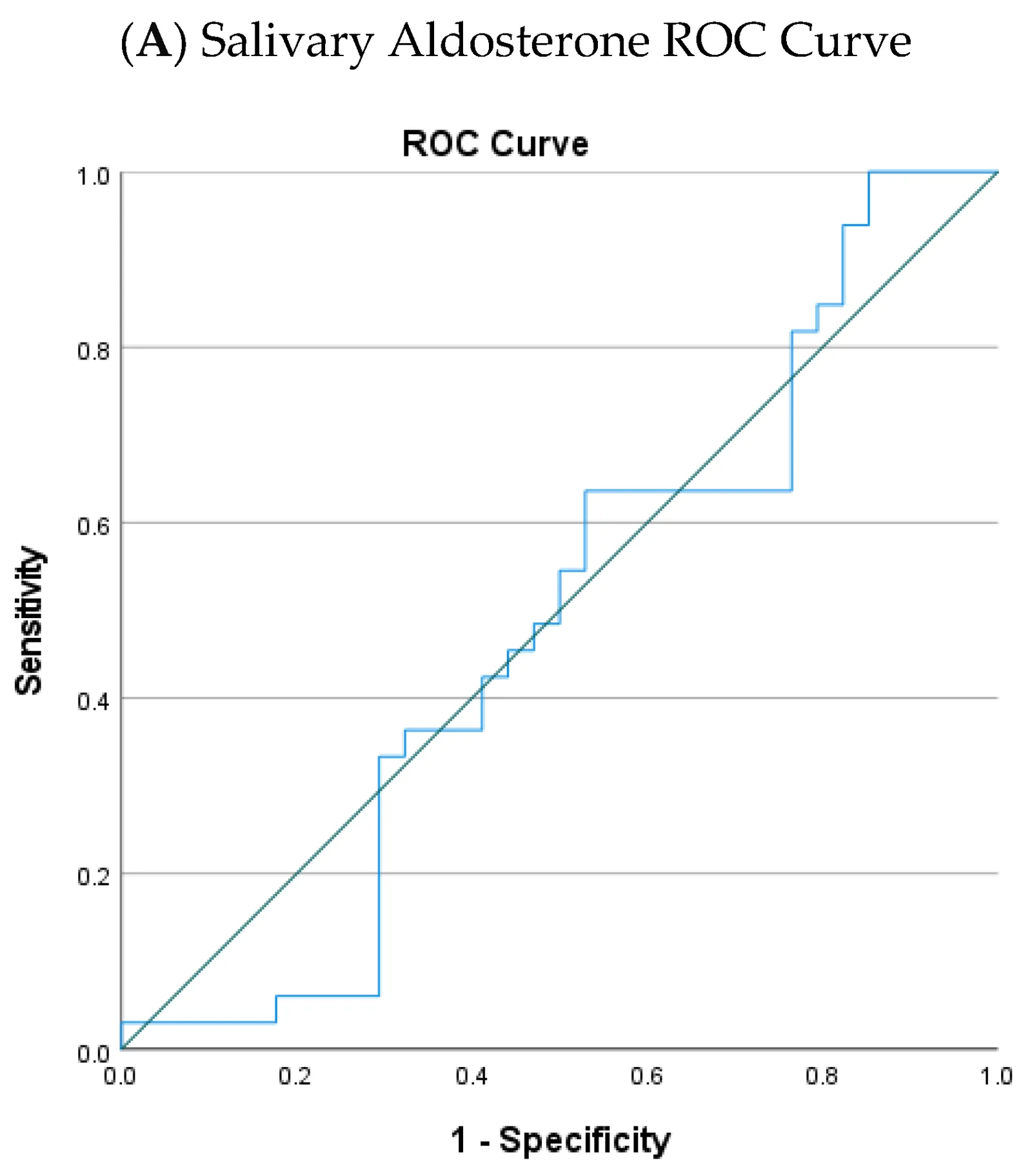

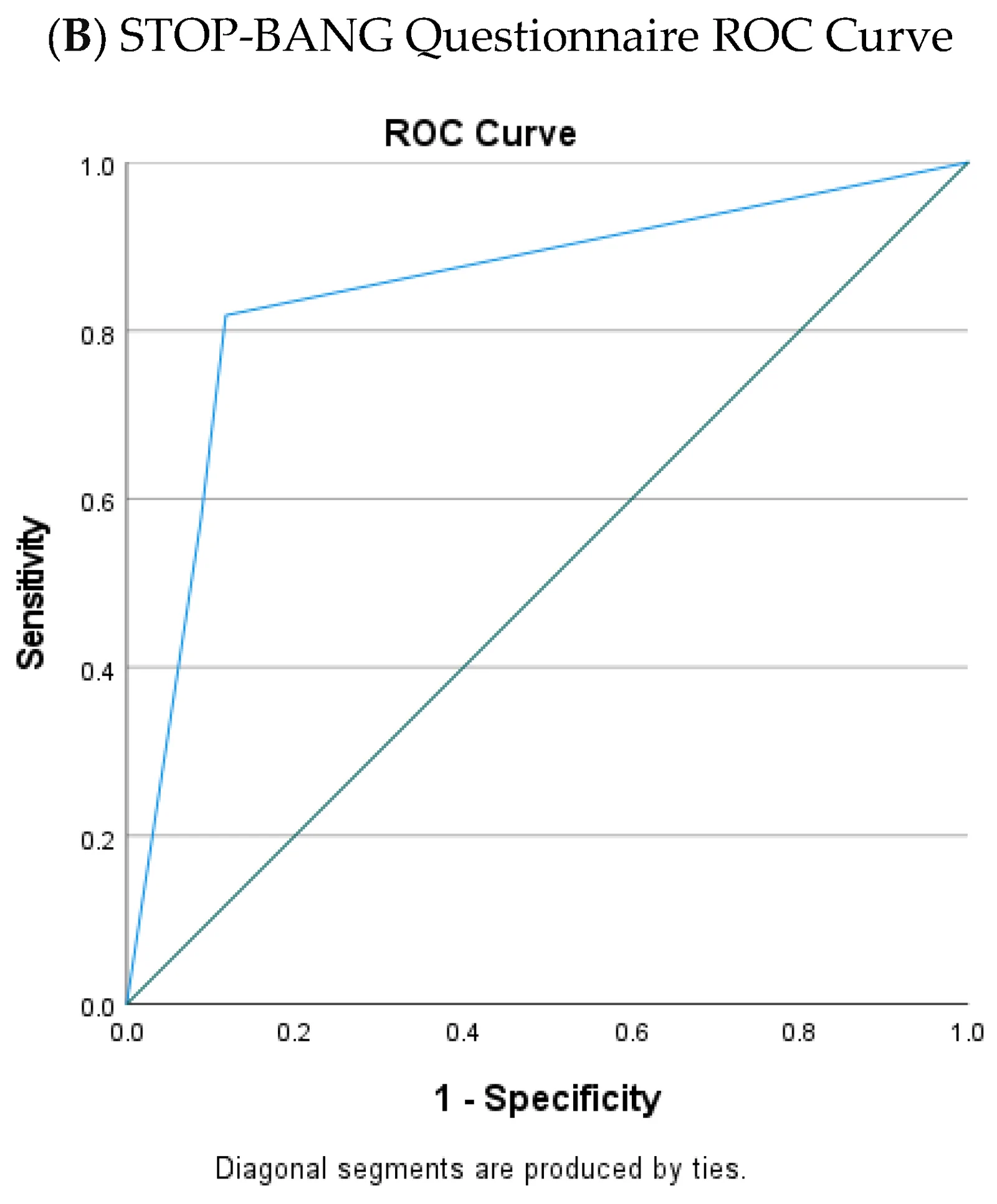
Receiver operating characteristic (ROC) curves for (**A**) salivary aldosterone and (**B**) STOP-BANG questionnaire for identifying moderate-to-severe OSA (AHI ≥ 15 events/h). OSA, obstructive sleep apnea.

Receiver Operating Characteristic (ROC) analysis demonstrated that salivary aldosterone had poor discriminative ability for identifying moderate-to-severe OSA (Area Under the Curve; AUC = 0.484, 95% CI 0.342–0.626), consistent with near-chance performance (Figure 2A). The STOP-BANG questionnaire demonstrated substantially superior performance (AUC = 0.848, 95% CI 0.748–0.948; Figure 2B).

Paired comparisons revealed comparable sensitivity between the two tools in the moderate-to-severe OSA group (McNemar *p* = 1.000). However, STOP-BANG demonstrated significantly higher specificity than salivary aldosterone among healthy or mild OSA participants (*p* = 0.003) and significantly higher overall diagnostic accuracy (*p* = 0.041).

## 3. Discussion

This study investigated salivary aldosterone as a non-invasive biomarker for OSA and factors modulating its levels. Three principal findings emerged: (1) salivary aldosterone did not differ across OSA severity groups and showed poor diagnostic accuracy as a standalone screening tool; (2) age was the only independent predictor in the adjusted model; and (3) MAP significantly moderated the relationship between OSA severity and salivary aldosterone, with elevated MAP amplifying aldosterone secretion selectively in moderate-to-severe OSA.

### 3.1. Absence of Group Differences in Salivary Aldosterone 

Salivary aldosterone did not differ across OSA severity groups and is consistent with previous plasma-based studies that similarly reported no significant aldosterone elevation in OSA patients without concurrent hypertension or significant cardiovascular comorbidities [23,24,25]. This suggests that OSA alone may not constitute a sufficient stimulus for clinically meaningful RAAS activation in normotensive individuals. In contrast, studies that reported elevated aldosterone typically involved resistant hypertension or metabolic syndrome [18,19], implying that RAAS activation may be contingent upon additional cardiometabolic burden. In the present cohort, participants generally had normal or mildly elevated blood pressure with no cases of resistant hypertension, which likely attenuated potential between-group differences. Additional contributors include variability in antihypertensive medication use and unaccounted dietary sodium intake, both of which are known to influence aldosterone levels [17]. Furthermore, OSA is a phenotypically heterogeneous disorder, and the pathophysiological mechanisms underlying aldosterone dysregulation may differ across OSA subtypes [26].

### 3.2. Age as an Independent Predictor of Salivary Aldosterone

Age emerged as the only significant independent predictor of salivary aldosterone in the adjusted model, with higher age associated with lower aldosterone. This aligns with evidence of age-related decline in RAAS responsiveness [27], which may be further compounded by reduced salivary gland function in older individuals [28]. Thus, salivary aldosterone may reflect both systemic RAAS activity and glandular secretory capacity, with the latter diminishing with age. This has important implications for the interpretation of salivary aldosterone in older OSA populations, where the biomarker’s signal may be attenuated independently of disease severity. Stratification by age and OSA phenotype in future studies may help identify subgroups—such as younger adults or those with obesity-related OSA—in whom salivary aldosterone retains greater diagnostic and mechanistic value [14].

### 3.3. MAP as a Moderator of the OSA–Aldosterone Relationship 

The most novel finding was the moderating effect of MAP on the relationship between OSA severity and salivary aldosterone. In moderate-to-severe OSA, higher MAP was associated with substantially elevated aldosterone, while this relationship was absent in the healthy or mild OSA group. This pattern is mechanistically plausible: OSA induces intermittent hypoxia and chronic sympathetic overactivation, which promotes renin release and RAAS stimulation [15,29]. In individuals with elevated MAP, RAAS activation may be amplified through heightened renal perfusion pressure sensitivity and vascular reactivity [30,31]. Together, these findings suggest that MAP acts as a biological amplifier of OSA-mediated RAAS activation: without elevated blood pressure, the degree of intermittent hypoxia produced by OSA may be insufficient to generate detectable salivary aldosterone elevation. Clinically, salivary aldosterone may have greater utility as a mechanistic and prognostic marker in the subgroup of OSA patients with elevated or borderline MAP, potentially aiding in early identification of those at heightened risk for hypertension-mediated volume overload and cardiovascular complications.

### 3.4. Diagnostic Performance of Salivary Aldosterone and STOP-BANG 

The poor discriminative ability of salivary aldosterone contrasts sharply with the clinically acceptable performance of the STOP-BANG questionnaire, which demonstrated significantly higher specificity and overall accuracy, making it more effective at ruling out non-OSA individuals. This is consistent with the established performance characteristics of STOP-BANG across diverse clinical populations [32]. The sensitivity of STOP-BANG observed in this study was somewhat lower than in previous reports, likely attributable to the stricter risk classification threshold applied, which increased specificity at the cost of sensitivity. Salivary aldosterone, while lacking standalone diagnostic value, may still contribute to multimodal diagnostic models or serve as a mechanistic indicator in clinical research contexts.

### 3.5. Limitations 

Several limitations warrant consideration. The relatively small sample size (*n* = 67), single-center design, and limited number of participants with mild OSA (*n* = 4) may have reduced statistical power for subgroup comparisons and diagnostic accuracy analyses. Therefore, the diagnostic performance findings and subgroup interpretations should be considered exploratory and require validation in larger, multicenter cohorts. Second, different diagnostic modalities were used for OSA assessment between groups. OSA participants were diagnosed using in-laboratory PSG, whereas healthy controls were screened using Level 3 home sleep apnea testing (HSAT). Because HSAT estimates respiratory events based on recording time rather than true total sleep time, it may underestimate the AHI compared with PSG. Therefore, some participants classified as healthy may have had undiagnosed mild OSA, resulting in potential misclassification of the control group. This potential misclassification may have influenced the observed differences in salivary aldosterone levels and the estimated diagnostic performance of salivary aldosterone. Future studies using PSG for both control and OSA groups are warranted to confirm these findings.

Third, although participants with medical conditions known to affect aldosterone secretion were excluded, residual confounding related to medications affecting the RAAS pathway cannot be completely excluded. Medication histories were collected, and RAAS-related medication use was reported; however, the number of participants receiving RAAS-blocking medications was small, limiting the feasibility of including medication use as an additional covariate in the regression models. Future studies with larger cohorts and more comprehensive medication data are needed to clarify the independent relationship between OSA, blood pressure regulation, and salivary aldosterone levels.

In addition, MAP is derived from a single seated blood pressure measurement and may not fully reflect the dynamic blood pressure profile in individuals with OSA, particularly the nocturnal blood pressure surges that commonly occur during sleep. Although MAP was included as a covariate and examined as a potential effect modifier in the present study, the clinical implications of this finding should be interpreted cautiously. Furthermore, given the relatively small sample size, the observed moderating effect of MAP should be considered exploratory and requires validation in larger, adequately powered studies. Future studies incorporating 24-h ambulatory blood pressure monitoring are warranted to better characterize nocturnal blood pressure variability and its relationship with aldosterone regulation in OSA.

Confounders including antihypertensive medication use, dietary sodium intake, and unmeasured comorbidities were not fully controlled. Finally, age-related decline in salivary gland function may have confounded aldosterone measurements in older participants.

## 4. Materials and Methods

### 4.1. Study Design and Participants

This cross-sectional study was conducted at the Faculty of Dentistry, Universiti Malaya, with OSA diagnoses established at the OSA Combined Clinic of Universiti Malaya Medical Center (UMMC). Participants were recruited by consecutive convenience sampling during routine dental visits between July 2024 and February 2025. Ethical approval was obtained from the Research Ethics Committee of the Faculty of Dentistry, Universiti Malaya (reference: DF CD 2411/0033 [P]), and written informed consent was obtained from all participants.

Eligible participants were adults aged ≥ 18 years who either had a new OSA diagnosis (within 6 months) confirmed by in-laboratory PSG, or healthy individuals without OSA (AHI < 5 events/h) confirmed by Level 3 HSAT. Exclusion criteria included night shift work; current or prior OSA treatment (e.g., continuous positive airway pressure or upper airway surgery); medical conditions affecting aldosterone secretion (including adrenal disorders, chronic kidney disease, or uncontrolled hypertension defined as systolic blood pressure ≥ 180 mmHg, diastolic blood pressure ≥ 110 mmHg, or requiring ≥ 3 antihypertensive agents); pregnancy; and irregular or reduced menstrual cycles.

Eight participants were excluded due to salivary aldosterone concentrations outside the enzyme-linked immunosorbent assay (ELISA) quantifiable range, resulting in a final sample of 67 participants.

### 4.2. Reference Standard and OSA Classification

OSA diagnosis in patients referred to the OSA Combined Clinic was established using overnight type-I PSG, which served as the reference standard for OSA assessment. The AHI was calculated according to standard scoring criteria and used to classify OSA severity.

Participants were initially classified according to AHI into healthy controls (AHI < 5 events/h; *n* = 30), mild OSA (AHI 5–14.9 events/h; *n* = 4), moderate OSA (AHI 15–29.9 events/h; *n* = 14), and severe OSA (AHI ≥ 30 events/h; *n* = 19). Healthy participants were screened using a Level 3 HSAT device (Belun Ring Platform). For the primary analyses, participants were classified a priori into two groups: healthy or mild OSA (*n* = 34) and moderate-to-severe OSA (*n* = 33). This approach was selected to reflect clinically relevant differences in OSA severity and to ensure sufficient sample size for statistical comparisons.

### 4.3. Measurement

#### 4.3.1. Salivary Aldosterone Measurement

Salivary aldosterone concentration was considered the index test. Unstimulated saliva samples were collected between 9:00 AM and 12:00 PM after participants rested in the supine position for approximately one hour. Samples were centrifuged and stored at −80 °C until analysis.

Aldosterone concentrations were measured using an ELISA kit according to the manufacturer’s instructions. Laboratory personnel performing the aldosterone assays were blinded to participants’ OSA status.

#### 4.3.2. Clinical Assessment and Covariates

Predictor variables included age, BMI, NC, and MAP. Age was obtained from records; BMI was calculated from measured height and weight; NC was measured at the cricothyroid membrane; and MAP was calculated as (SBP + 2 × DBP)/3 from seated blood pressure readings obtained using a calibrated automated sphygmomanometer.

Medication history was obtained through participant self-report or medical records during the clinical assessment. Current medications were reviewed and categorized according to their potential relevance to aldosterone regulation, including antihypertensive medications, angiotensin II receptor blocker (ARB), and statins. These variables were selected based on known associations with OSA severity and aldosterone physiology, with MAP additionally examined as an effect modifier.

#### 4.3.3. STOP-BANG Questionnaire Assessment

STOP-BANG questionnaires were completed on the same day, categorized into low (0–2), moderate (3–4), and high risk (5–8), with a threshold of ≥5 used to identify moderate-to-severe OSA in diagnostic analyses [32].

### 4.4. Sample Size Estimation

The minimum sample size for multiple regression was estimated using the 20 + 5 k rule, requiring at least 45 participants; the final sample (*n* = 67) met this requirement, although ROC analyses were exploratory due to limited power.

### 4.5. Statistical Analysis

Statistical analyses were performed using IBM SPSS Statistics (Version 27.0; IBM Corp., Armonk, NY, USA). Normality was assessed using the Shapiro–Wilk test. Given the non-normal distribution of salivary aldosterone, group comparisons were performed using the Mann–Whitney U test, and results are reported as median [interquartile range, IQR]. Categorical variables were compared using the chi-square test or Fisher’s exact test, as appropriate. Fisher’s exact test was applied when expected cell counts were small.

Salivary aldosterone was log-transformed prior to regression analyses to improve distributional properties. Associations between OSA severity and salivary aldosterone were analyzed using GLM with salivary aldosterone as the dependent variable, adjusting for age, BMI, NC, and MAP.

A supplementary multiple linear regression analysis was performed with AHI as a continuous predictor to further evaluate the association between OSA severity and salivary aldosterone levels, with adjustment for the same covariates.

The moderating effect of MAP on the relationship between OSA severity and log-transformed aldosterone was examined using the PROCESS macro (Model 1), adjusting for age, BMI, and NC as covariates.

Diagnostic accuracy of salivary aldosterone and the STOP-BANG questionnaire for detecting moderate-to-severe OSA was evaluated using ROC analysis. For binary classification of salivary aldosterone in the McNemar comparison, the optimal cut-off was determined using the Youden index (J = sensitivity + specificity − 1) derived from the ROC curve. Paired comparisons of sensitivity and specificity between salivary aldosterone and STOP-BANG were assessed using McNemar’s test.

All statistical tests were two-tailed, and *p* < 0.05 was considered statistically significant.

## 5. Conclusions

Salivary aldosterone did not differ across OSA severity groups and showed poor standalone diagnostic performance for OSA screening. Age independently and inversely predicted salivary aldosterone, consistent with age-related RAAS decline and reduced salivary gland function in older individuals. Critically, MAP significantly moderated the relationship between OSA severity and salivary aldosterone, with a clinically meaningful aldosterone–MAP association observed exclusively in individuals with moderate-to-severe OSA. These findings indicate that salivary aldosterone is unlikely to serve as a reliable screening biomarker in unselected OSA populations but may have selective mechanistic and prognostic relevance in OSA patients with elevated blood pressure. Future larger, multicenter, longitudinal studies incorporating 24-h ambulatory blood pressure monitoring and stratified analyses by age and OSA phenotype are warranted to clarify these relationships.

## Figures and Tables

**Table 1:** Sociodemographic and clinical characteristics of study participants.

Characteristics	Healthy and Mild OSA (*n* = 34)	Moderate-to-Severe OSA (*n* = 33)	Total (*n* = 67)
Age, years	27.4 ± 7.0	45.1 ± 15.5 *	36.1 ± 14.9
Male, *n* (%)	17 (50)	16 (48.5)	33 (49.3)
Female, *n* (%)	17 (50)	17 (51.5)	34 (50.7)
BMI, kg/m^2^	22.0 ± 5.0	29.6 ± 6.6 *	25.7 ± 6.9
SBP, mmHg	115.9 ± 17.6	129.5 ± 13.9 *	122.6 ± 17.3
DBP, mmHg	75.2 ± 12.7	81.5 ± 11.7 *	78.3 ± 12.6
MAP, mmHg	88.7 ± 14.1	97.5 ± 11.6 *	93.0 ± 13.6
NC, cm	35.3 ± 3.9	38.8 ± 4.2 *	37.1 ± 4.4
ODI, events/h	3.4 ± 3.0	31.1 ± 25.2 *	17.2 ± 22.8
Lowest SpO_2_, %	87.5 ± 6.5	78.7 ± 11.2 *	83.1 ± 10.2
Comorbidities			
Hypertension, *n* (%)	0 (0)	11 (33.3)	11 (33.3)
Dyslipidemia, *n* (%)	0 (0)	3 (9.1)	3 (9.1)
Cardiovascular disease, *n* (%)	0 (0)	1 (3.0)	1 (3.0)
Cerebrovascular disease, *n* (%)	0 (0)	1 (3.0)	1 (3.0)
Medication use			
Antihypertensive medication, *n* (%)	0 (0)	9 (27.3)	9 (27.3)
ARB, *n* (%)	0 (0)	2 (6.1)	2 (6.1)
Statin use, *n* (%)	0 (0)	3 (9.1)	3 (9.1)

Data are presented as mean ± SD or *n* (%). * Significantly different from the healthy and mild OSA group (*p* < 0.05). Abbreviations: OSA, obstructive sleep apnea; BMI, body mass index; SBP, systolic blood pressure; DBP, diastolic blood pressure; MAP, mean arterial pressure; NC, neck circumference; ODI, oxygen desaturation index; SpO_2_, peripheral oxygen saturation; ARB, Angiotensin II Receptor Blocker.

**Table 2:** Salivary aldosterone concentrations between OSA groups.

Variable	Healthy or Mild OSA (*n* = 34)	Moderate-to-Severe OSA (*n* = 33)	*p*-Value
Salivary aldosterone, pg/mL Median [IQR]	68.35 (41.15–510.50)	68.54 (34.98–293.30)	0.821

Data are presented as median [interquartile range]. Group comparison by Mann–Whitney U test. Abbreviations: OSA, obstructive sleep apnea; IQR, interquartile range.

**Table 3:** Generalized linear model parameter estimates for factors associated with salivary aldosterone levels.

Predictor	Coefficient (B)	95% CI	*p*-Value
OSA severity (healthy/mild vs. moderate-to-severe)	0.02	[−0.935, 0.975]	0.968
Age (years)	−0.035	[−0.062, −0.009]	0.010 *
BMI (kg/m^2^)	−0.002	[−0.070, 0.065]	0.948
NC (cm)	0.035	[−0.057, 0.128]	0.454
MAP (mmHg)	0.01	[−0.023, 0.042]	0.561

* *p* < 0.05. Multiple linear regression; dependent variable: log-transformed salivary aldosterone. Abbreviations: OSA, obstructive sleep apnea; BMI, body mass index; NC, neck circumference; MAP, mean arterial pressure; CI, confidence interval.

## Data Availability

The raw data supporting the findings of this study are publicly available in the Figshare repository at https://doi.org/10.6084/m9.figshare.31124650.

## Supplementary Materials

The following supporting information can be downloaded at https://www.mdpi.com/article/10.3390/clockssleep8030047/s1.

## Abbreviations

The following abbreviations are used in this manuscript:

OSA	Obstructive sleep apnea
RAAS	Renin–angiotensin–aldosterone system
MAP	Mean arterial pressure
AHI	Apnea–hypopnea index
GLM	Generalized linear model
ROC	Receiver operating characteristic
AUC	Area under the curve
PSG	Polysomnography
HSAT	Home sleep apnea testing
BMI	Body mass index
NC	Neck circumference
SBP	Systolic Blood Pressure
DBP	Diastolic Blood Pressure
ELISA	Enzyme-linked immunosorbent assay
ODI	Oxygen desaturation index
ARB	Angiotensin II receptor blocker
