# Supplementary material for: Salivary Aldosterone as a Biomarker for Obstructive Sleep Apnea: Diagnostic Performance and the Moderating Role of Mean Arterial Pressure—A Cross-Sectional Study
Source: Clocks Sleep. 2026 Aug 17;8(3):47. doi: 10.3390/clockssleep8030047 (PMC13510994; doi:10.3390/clockssleep8030047)
Supplement: Supplementary file 1 [file clockssleep-08-00047-s001.zip › clockssleep-4410667-supplementary.pdf]

Supplementary Table S1. Association between apnea–hypopnea index and salivary aldosterone levels using multiple linear regression analysis

Predictor	Coefficient (B)	95% CI	p-value
AHI (events/h)	-0.004	[-0.012, 0.003]	0.252
Age (years)	-0.005	[-0.016, 0.006]	0.364
BMI (kg/m2)	0.005	[-0.025, 0.036]	0.722
NC (cm)	0.011	[-0.033, 0.054]	0.623
MAP (mmHg)	0.004	[-0.008, 0.017]	0.505
